# An integrated multi-factor decision model for personalized NIPT timing using BMI stratification

**DOI:** 10.1038/s41598-026-48094-1

**Published:** 2026-04-16

**Authors:** Xiaoya Luo, Jiali Wang, Yanlan Yang, Cuihua He

**Affiliations:** 1 School of Integrated Circuits, Chengdu Technological University, Chengdu, 611730 Sichuan China; 2 School of Data Science, Chengdu Technological University, Chengdu, 611730 Sichuan China

**Keywords:** Non-invasive prenatal testing (NIPT), Multi-dimensional modeling, Nonlinear mixed-effects model, Joint optimization model, Detection of chromosomal abnormalities in female fetuses, Biomarkers, Computational biology and bioinformatics, Genetics, Medical research

## Abstract

Non-invasive prenatal testing (NIPT) is a prenatal screening technique that analyzes cell-free fetal DNA in maternal blood plasma, widely used for early detection of fetal chromosomal abnormalities such as trisomy 21, 18, and 13. To optimize personalized testing timing and improve the detection accuracy of chromosomal abnormalities in female fetuses, this study developed a data-driven multifactorial modeling framework. The study population consisted of 1,687 pregnant women from a certain region. Correlation analysis and a nonlinear mixed-effects model (M4) were employed to quantify the relationships among gestational age, body mass index (BMI), and Y-chromosome concentration. The model results showed a positive correlation between gestational age and Y-chromosome concentration, while BMI and height were significantly negatively correlated with Y-chromosome concentration, providing statistical support for subsequent BMI-based stratification. Building on this, the study further predicted the earliest possible gestational week at which fetal DNA concentration reaches the detection threshold for each pregnant woman. A joint optimization model for BMI grouping and testing timing was constructed to determine the optimal testing time for each group, and the interaction between BMI and gestational age was validated using K-means clustering incorporating multiple factors. The model results indicated that the M4 model exhibited the best goodness of fit. As BMI increased, the recommended testing time was postponed from 13.8 weeks to 21.5 weeks. Additionally, to address the issues of limited data and the absence of Y-chromosome signals in female fetuses, this study systematically compared decision tree, random forest, and support vector machine (SVM) models, constructing a multi-feature fusion-based classification system. In the task of classifying chromosomal abnormalities in female fetuses, the SVM model performed the best, with X-chromosome concentration and GC content identified as key discriminatory features. The comprehensive multifactorial model proposed in this study can effectively predict personalized NIPT timing, help reduce the risk of testing failure, enhance the detection performance for chromosomal abnormalities in female fetuses, and thereby provide scientific basis and methodological support for the clinical application of NIPT.

**Supplementary Information:**

The online version contains supplementary material available at 10.1038/s41598-026-48094-1.

## Introduction

With the continuous optimization of China’s fertility policies, the support system has evolved from measures focused on “reducing burdens” – such as economic subsidies, childcare service provision, and workplace rights protection – to a new stage centered on improving the quality of the newborn population through medical technological innovation. Non-invasive prenatal testing (NIPT)^1^ represents a breakthrough in this stage. Due to its significant advantages of “high detection accuracy and low invasiveness^2,3^,” it has rapidly replaced traditional screening methods and become a core component of modern prenatal screening. According to data from the *China Birth Defects Prevention Report (2012)*^4^, the incidence of birth defects in China is approximately 5.6%, with chromosomal abnormalities being a major contributing factor. NIPT demonstrates excellent sensitivity in screening for common chromosomal disorders^5^, its detection sensitivity for Patau syndrome (Trisomy 13) reaches 85%–90%, for Edwards syndrome (Trisomy 18) it is typically around 95%, and for Down syndrome (Trisomy 21) it is as high as approximately 99%. This provides reliable technological assurance for reducing the risk of birth defects and improving birth quality.

Existing studies have confirmed that the effectiveness of NIPT is closely related to various factors, including the fetal DNA concentration, gestational week at testing, and maternal physiological characteristics^6^. The performance of NIPT is highly dependent on the proportion of cell-free fetal DNA (cffDNA)in maternal peripheral blood, known as the fetal fraction (FF). Clinical practice typically requires an FF $\ge$ 4% to ensure result reliability. Maternal Body Mass Index (BMI) is a key physiological factor influencing FF^7^. Pregnant women with a high BMI have a higher total concentration of cell-free DNA in their peripheral blood, leading to a “dilution” of the cffDNA proportion and a significant decrease in FF. Studies have shown that among pregnant women with a BMI $\ge$ 30 kg/m^2^, the proportion with FF < 4% can reach 15%–20%, which is 3–5 times higher than that in the normal BMI group (BMI 18.5–24.9). This phenomenon directly leads to an increased false-positive rate, a higher retesting rate, and even “no-call” results in the high-BMI population. However, current clinical NIPT testing typically employs a “uniform testing window” (mostly 10–20 weeks of gestation), which is designed based on population data indicating “stabilization of FF after 10 weeks of gestation” but does not account for individual physiological differences. The limitations of this uniform window are particularly pronounced for pregnant women with a high BMI: testing early at 10–12 weeks carries a higher risk of insufficient FF, while delaying testing beyond 20 weeks may increase FF but also heightens maternal psychological anxiety and complicates arrangements for potential mid-term termination of pregnancy^8^. To date, most clinical guidelines merely suggest that “pregnant women with a high BMI may consider delayed testing^9,10^” without providing a specific, quantifiable BMI–gestational age matching scheme, leaving clinical practice lacking a clear quantitative basis. Regarding the determination of abnormalities in female fetuses, the absence of the Y chromosome in female fetuses means existing methods predominantly rely on single-chromosome Z-score analysis, overlooking the synergistic role of multi-dimensional features such as X chromosome indicators, GC content, and read quality. These research gaps highlight significant shortcomings in the current NIPT testing framework concerning personalized adaptation and integrated multi-factor analysis.

Based on the aforementioned status, this study proceeds from actual clinical needs to address the core issues of the lack of personalized criteria for male fetal testing timing and the single-dimensional assessment for female fetal abnormalities. By constructing a multi-factor integrated model, it explores the regulatory mechanisms of key factors such as gestational age and BMI on testing efficacy, establishes optimal testing time points for different subgroups, and builds a multi-feature fusion-based system for assessing female fetal abnormalities. This work provides a scientific basis for enhancing the precision and personalization of NIPT testing.

## Model construction

### Source of data

The data for this study were obtained from the publicly available dataset for Problem C of the 2025 “Higher Education Society Cup” National Mathematical Contest in Modeling. The dataset spans from March 2023 to July 2024 and comprises 1,687 test records, including 1,082 from male fetuses and 605 from female fetuses.

To identify optimal testing time points for specific subgroups, a comprehensive analysis of key indicators in the dataset is required. These include maternal age, gestational week, body mass index (BMI), genomic GC content, and Z-score, among others. Established evidence indicates that these metrics are critical for determining individualized optimal testing timing. Their systematic analysis helps minimize potential risks throughout the testing process.

### Date pre-processing

To ensure the consistency of units and facilitate subsequent modeling, this paper first precisely converts the character type ”gestational age at detection”. For the number of days less than one week, it is converted as a whole into a decimal part of a continuous value in weeks. The calculation formula is

1 $$\begin{aligned} W_1=W_0+\frac{Day}{7} \end{aligned}$$

Here,$W_1$ represents the consecutive number of weeks,$W_0$ represents the number of weeks,and *Day* represents the number of days.

### Construction of correlation model between gestational age, BMI, and Y-chromosome concentration

Practice has shown a close association between fetal Y-chromosome concentration and maternal gestational age as well as body mass index (BMI). To further investigate this relationship, this study first conducted a descriptive statistical analysis of the main variables in the dataset to reveal the data distribution characteristics and potential relationships between variables, providing an empirical basis for subsequent model selection. Subsequently, the Spearman correlation coefficient was employed to analyze the correlations between continuous variables, as this method can better capture the rank correlation trends between variables. A heatmap of correlation coefficients was generated to visually present these relationships. During the modeling phase, four models with progressively increasing complexity were constructed, ranging from linear to nonlinear and from no random effects to the inclusion of random effects. Finally, the optimal model was selected based on a comparison of statistical criteria.

#### Visual analysis

First, descriptive statistical analysis^11^ was performed on the main variables in the dataset to reveal their distribution characteristics and provide preliminary insights into the relationships between variables, thereby offering an empirical basis for subsequent model selection.

**Table 1:** Descriptive analysis of main variables and pearson correlation coefficient matrix.

Variable	Minimum	Maximum	Mean	Standard deviation	Pearson correlation coefficient
Y-Chromosome Concentration	0.01	0.23	0.08	0.03	1
Gestational Age (weeks)	11.00	29.00	16.85	4.08	0.13
Maternal BMI (kg/m^2^)	20.70	45.88	32.15	2.97	-0.15

**Fig. 1:**
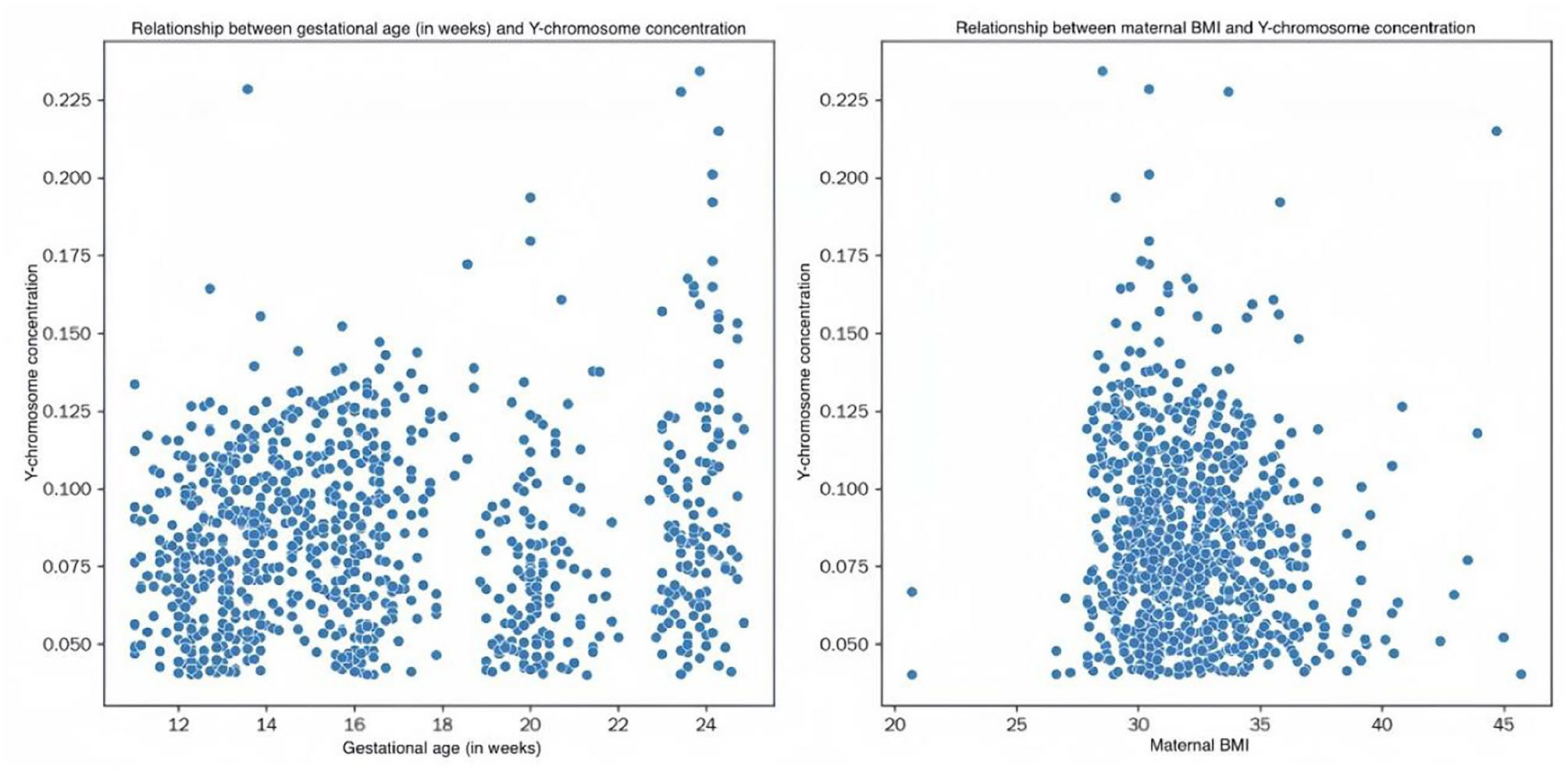
Scatter plot of Y-chromosome concentration versus gestational age and maternal BMI.

Based on the results in Table 1, the correlation coefficient between Y-chromosome concentration and gestational age is 0.13, and that between Y-chromosome concentration and maternal BMI is -0.15, indicating a very weak linear correlation between these variables. Furthermore, to more intuitively explore potential nonlinear trends or distribution characteristics among the variables, a scatter plot of Y-chromosome concentration against gestational age and BMI was generated as shown in Fig. 1.

As shown in Fig. 1, data points representing Y-chromosome concentration across gestational weeks are widely scattered, indicating a very weak linear correlation between the two. When maternal BMI is approximately in the range of 25–35 kg/m^2^, the distribution of Y-chromosome concentration appears relatively concentrated. However, overall, as maternal BMI increases, Y-chromosome concentration exhibits a fluctuating downward trend, which does not indicate a stable linear relationship between the two variables.

#### Exploration of nonlinear associations

**Fig. 2:**
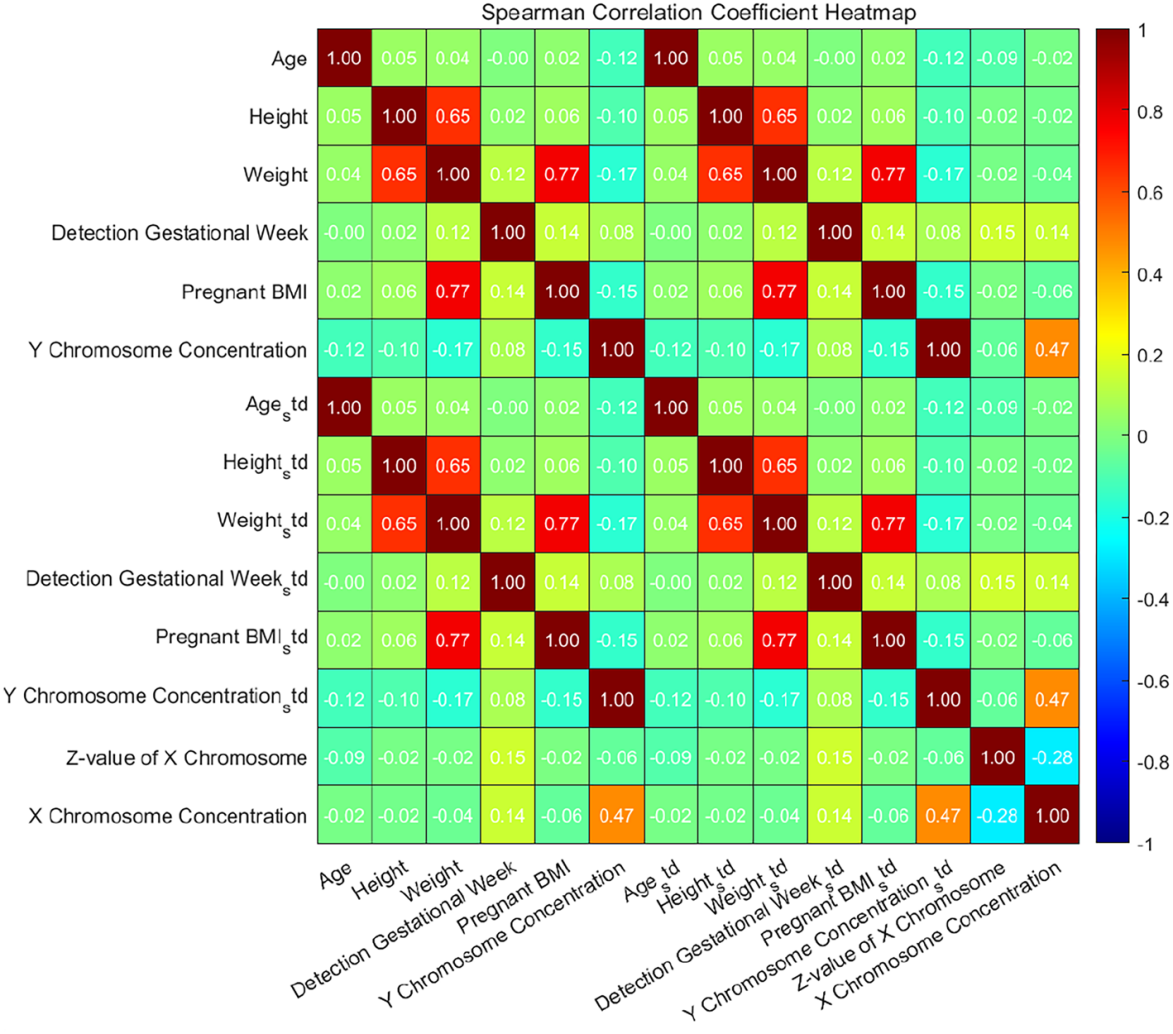
Spearman correlation coefficient heatmap.

Based on the Pearson correlation coefficient analysis and scatter plot analysis^12^ in Table 1, the linear association between Y chromosome concentration and gestational age at testing, as well as maternal BMI, was extremely weak. There may be nonlinear trends among the variables that cannot be captured by linear models. Further, this study utilized the characteristic of the Spearman correlation coefficient^13^, which is more suitable for capturing rank correlation trends between variables, to analyze the continuous variables, and the Spearman correlation coefficient heatmap is shown in Fig. 2.

As shown in Fig. 2, the Y chromosome concentration had a moderate positive correlation with the X chromosome concentration and a moderate negative correlation with the number of blood draws. The absolute values of its correlation coefficients with all other variables were less than 0.2, indicating extremely weak associations. Therefore, it is necessary to further explore this issue by constructing a hierarchical and progressive model system.

#### Construction of a hierarchical progressive model

A hierarchy of four models with increasing complexity from linear to nonlinear, and from no random effects to incorporating random effect was constructed. The optimal model was subsequently selected based on statistical criteria.

The multiple linear regression model ($M_1$)^14^ serves as the baseline. It was assumed that there is a linear relationship between the Y chromosome concentration and gestational age at testing (*w*) as well as maternal BMI (*b*), and that all observation points are mutually independent. Its mathematical formula can be expressed as

2 $$\begin{aligned} Y_{ij} = \beta _0 + \beta _1 w_{ij} + \beta _2 b_{ij} + \delta _{ij}, \end{aligned}$$

where $Y_{ij}$ and $w_{ij}$ represent the Y-chromosome concentration and the gestational week at the *j*-th examination for the *i*-th pregnant woman, respectively. $b_{ij}$ denotes the maternal BMI at the *j*-th examination for the *i*-th woman. $\beta _0$ is the intercept term, $\beta _1$ and $\beta _2$ are the coefficients for the linear effects of gestational age and maternal BMI, respectively. $\delta _{ij}$ is the random error term following a normal distribution $N(0, \sigma ^2)$.

Building upon this foundation, a nonlinear polynomial regression model $(M_2)$ was constructed, incorporating higher-order terms of the independent variables to capture potential nonlinear relationships. Its mathematical formulation is as follows

3 $$\begin{aligned} Y_{ij} = \beta _0 + \sum _{k=1}^{m} \beta _k w_{ij}^k + \sum _{l=1}^{h} \gamma _l b_{ij}^l + \delta _{ij}, \end{aligned}$$

where $\beta _k$ and $\gamma _l$ are the regression coefficients for the *k*-th order term of gestational age and the *l*-th order term of BMI, respectively, and *m* and *h* are the predefined highest degrees of the polynomials.

To account for the effects of individual variations among pregnant women, a linear mixed-effects model $(M_3)$ was further developed by introducing random effects terms that represent individual differences, based on Eq. (2). Its mathematical expression is

4 $$\begin{aligned} Y_{ij} = \left( \beta _0 + u_{0i} \right) + \beta _1 w_{ij} + \beta _2 b_{ij} + \delta _{ij}, \end{aligned}$$

where $u_{0i}$ is the random intercept for the *i*-th pregnant woman, accounting for individual-level variation.

Finally, by integrating the structural strengths of the $M_1$, $M_2$, and $M_3$ models, a nonlinear mixed-effects model $(M_4)$ was formulated to capture overall nonlinear trends through polynomial fixed effects while explaining individual-level variation through random effects. Its formula is

5 $$\begin{aligned} Y_{ij} = (\beta _0 + u_{0i}) + \sum _{k=1}^m \beta _k w_{ij}^k + \sum _{l=1}^h \gamma _l b_{ij}^l + \delta _{ij}. \end{aligned}$$

Statistical criteria were used to evaluate and screen the aforementioned models, so as to determine the necessity of upgrading the models from simple to complex forms. The presence of significant individual differences was formally verified by the likelihood ratio test, namely, whether the variance of the random effects was greater than zero.

First, the null and alternative hypotheses were established. The null hypothesis $H_0: \sigma _u^2 = 0$ posits that the variance of the random effects among individuals is zero, indicating no significant individual differences, in which case the simpler model $M_1$ without random effects would be appropriate. The alternative hypothesis $H_1: \sigma _u^2 > 0$ posits that the variance of the random effects is significantly greater than zero, indicating non-negligible individual differences, in which case the more complex model $M_3$ would be required.

Subsequently, the test statistic was constructed. The test statistic $\Lambda$ for the LRT is determined by comparing the log-likelihood values^15^ of the $M_1$ model under the null hypothesis and the $M_3$ model under the alternative hypothesis.

$$\Lambda = -2\ln \left( \frac{L\left( \hat{\theta }_0 \right) }{L\left( \hat{\theta }_1 \right) } \right) = -2\left( l_0 - l_1 \right) ,$$

where $L\left( \hat{\theta }_0 \right)$ and $l_0$ denote the likelihood function value and the log-likelihood value, respectively, for model $M_1$ at its maximum likelihood estimated parameters. $L\left( \hat{\theta }_1 \right)$ and $l_1$ represent the corresponding likelihood function value and log-likelihood value for model $M_3$.

If the null hypothesis $H_0$ holds true, the test statistic $\Lambda$ approximately follows a chi-squared distribution with one degree of freedom, denoted as $\chi ^2(1)$. This is because the two models differ by only one parameter, $\sigma _u^2$. The actually observed value of $\Lambda$ is calculated, and the corresponding *p*-value is derived from this chi-squared distribution. If this *p*-value is smaller than the pre-defined significance level $\alpha$ (typically 0.05), the null hypothesis is rejected, indicating statistically significant individual differences.

Building on the hypothesis testing framework, the significance of fixed-effect parameters within each model is further assessed using *t*-tests or *z*-tests. For model selection, the root mean square error (*RMSE*)^16^ serves as the primary metric for comparing model performance across all candidates. It is defined as follows

6 $$\begin{aligned} RMSE = \sqrt{\frac{1}{N} \sum _{i=1}^{M} \sum _{j=1}^{n_i} \left( Y_{ij} - \hat{Y}_{ij} \right) ^2}, \end{aligned}$$

where *N* represents the total number of observations, and $\hat{Y}_{ij}$ denotes the model-predicted value for $Y_{ij}$. Due to its property of global comparability and its clear physical interpretation (i.e., the average prediction error of the model), RMSE was therefore adopted as the final criterion for selecting the optimal model.

### Joint optimization model for BMI stratification and testing timepoint

Clinical evidence has shown that maternal BMI is a primary factor affecting the earliest time at which fetal Y-chromosome concentration reaches or exceeds the 4% threshold. To reduce potential risks for pregnant women, this study established a reasonable BMI grouping for pregnancies carrying male fetuses, defining the corresponding BMI ranges and recommending optimal NIPT testing time points for each group, while further analyzing the influence of testing errors on the results. To this end, a two-fold optimization problem was constructed. First, given specific BMI groupings, the optimal testing time point minimizing risk within each group was determined. Second, the BMI group boundaries themselves were treated as optimization variables to evaluate the impact of different grouping schemes on overall risk, thereby simultaneously identifying both the “optimal BMI grouping” and its corresponding best testing time points.

#### Decision framework construction

Assume that pregnant women are stratified into *K* mutually exclusive groups based on BMI, with the $K-1$ cutoff points for BMI denoted as $b_k$, forming the decision vector $\boldsymbol{B}$. For each group *k* ($k \in \{1,2,\dots ,K\}$), a recommended gestational week for testing $W_k$ is determined, forming the decision vector $\boldsymbol{W}$. Within the feasible ranges of the decision variables $\boldsymbol{B}$ and $\boldsymbol{W}$, we seek the optimal combination that minimizes the global expected total potential risk $R_{\text {total}}(\boldsymbol{B},\boldsymbol{W})$, formulated as

7 $$\begin{aligned} \min _{\boldsymbol{B},\boldsymbol{W}} R_{\text {total}}(\boldsymbol{B},\boldsymbol{W}) = \sum _{k=1}^{K} P\left( \text {BMI} \in [b_{k-1}, b_k] \right) \cdot R_k(W_k), \end{aligned}$$

where $b_0$ is the minimum BMI value in the processed sample, and $b_K$ is the maximum BMI value $P\left( \text {BMI} \in [b_{k-1}, b_k] \right)$ represents the probability that BMI falls within the interval $[b_{k-1}, b_k]$; $R_k(W_k)$ denotes the potential risk faced by pregnant women in the *k*-th subgroup when undergoing NIPT testing at the recommended gestational week $W_k$.

To verify that the BMI stratification strategy offers a significant advantage over a single optimal timepoint applicable to all pregnant women, the following hypothesis test is constructed. Null hypothesis $H_0$: There is no difference in the global risk between the BMI stratification strategy and the non-stratified strategy, i.e.,

8 $$\begin{aligned} R_{\text {total}}\left( W_u'\right) - R_{\text {total}}\left( B', W'\right) = 0. \end{aligned}$$

Alternative hypothesis $H_1$: The BMI stratification strategy can significantly reduce the global risk, i.e.,

9 $$\begin{aligned} R_{\text {total}}\left( W_u'\right) - R_{\text {total}}\left( B', W'\right) > 0, \end{aligned}$$

where $R_{\text {total}}(W_u')$ denotes the global minimum risk obtained by a single decision variable *W* without stratification, and $R_{\text {total}}(\boldsymbol{B'},\boldsymbol{W'})$ denotes the global minimum risk corresponding to the stratified decision.

A nonparametric permutation test was employed to validate the risk reduction effect of the BMI stratification strategy. The observed risk reduction was calculated as $\Delta _{\text {obs}} = R_{\text {total}}(W_u') - R_{\text {total}}(\boldsymbol{B'},\boldsymbol{W'})$. The BMI labels of the samples were randomly shuffled multiple times, and the stratification and optimization procedures were repeated to obtain simulated risk reductions $\Delta _{\text {perm}}$. If $\Delta _{\text {obs}}$ fell within the top 5% of the empirical distribution formed by all $\Delta _{\text {perm}}$ (i.e., $p<0.05$), the null hypothesis $H_0$ was rejected, indicating that the BMI stratification strategy could significantly reduce the global risk.

10 $$\begin{aligned} R_k(W_k) = q \cdot \mathbb {E}\left[ R_{\text {f}}(W_k) \right] + (1-q) \cdot \mathbb {E}\left[ R_{\text {d}}(W_k) \right] , \end{aligned}$$

where $\mathbb {E}\left[ R_{\text {f}}(W_k) \right]$ is the expected test failure risk for group *k* (the expected risk of test failure due to the Y-chromosome concentration falling below the 4% threshold); $\mathbb {E}\left[ R_{\text {d}}(W_k) \right]$ is the expected delayed diagnosis risk for group *k* (the expected risk of diagnostic delay due to a late testing timepoint). $q \in (0,1)$ is the risk preference weight. Initially, *q* can be set to 0.5, and its value can be varied in subsequent analyses.

For $\mathbb {E}\big [R_{\text {f}}(W_k)\big ]$, the cause of test failure is that the Y-chromosome concentration at the testing timepoint $W_k$ does not reach 4%. Based on the $M_2$ model, the Y-chromosome concentration $Y_i(W_k)$ for any woman *i* in group *k* at gestational week $W_k$ can be expressed as

11 $$\begin{aligned} Y_i(W_k)=f(W_k, BMI_i)+\mu _{0i}+\delta _i, \end{aligned}$$

where $f(W_k, \text {BMI}_i)$ is the fixed-effects predicted mean determined by gestational week and BMI.

For a pregnant woman with a specific $\text {BMI}_i$, the probability of test failure is $P(Y_i(W_k) < 0.04)$. Since both $\mu _{0i}$ and $\delta _i$ follow normal distributions, $Y_i(W_k)$ also follows a normal distribution based on additivity. Letting the total variance be $\sigma ^2_{\text {total}}=\sigma ^2_U+\sigma ^2_\epsilon$, this probability can be precisely calculated using the standard normal cumulative distribution function $\Phi (\cdot )$. The expected test failure risk for group *k* is the average of the failure probabilities across all individuals within the group, expressed as

12 $$\begin{aligned} \mathbb {E}\big [R_f(W_k)\big ]=\frac{1}{N_k}\sum _{i_k\in \text {group}k}P\big (f(W_k, BMI_i)+Z_i<0.04\big ), \end{aligned}$$

that is

13 $$\begin{aligned} \mathbb {E}\big [R_f(W_k)\big ]=\frac{1}{N_k}\sum _{i_k\in \text {group}k}\Phi \left( \frac{0.04-f(W_k, BMI_i)}{\sigma _{\text {total}}}\right) . \end{aligned}$$

For $\mathbb {E}\big [R_d(W_k)\big ]$, this function increases with $W_k$ and exhibits an increasing rate of growth. Specifically, it can be defined as

14 $$\begin{aligned} \mathbb {E}\big [R_d(W_k)\big ]=R_d(W_k)={\left\{ \begin{array}{ll} 0, & W_k<12 \\ C_1(W_k-12), & 12\le W_k<27 \\ C_1(27-12)+C_2(W_k-27), & W_k\ge 27 \end{array}\right. } \end{aligned}$$

where $C_1$ and $C_2$ are preset penalty coefficients, representing the incremental risk per week of delay during the mid-term and late-term stages, respectively. To reflect the sharp increase in risk, it is set that $C_1 < C_2$.

#### Joint optimization model

To determine both the BMI stratification and the optimal testing timepoint, a dual optimization problem needs to be solved simultaneously: identifying the “optimal BMI grouping” and the best NIPT testing timepoint for each group. On one hand, given a BMI grouping, the testing timepoint that minimizes the risk within each group must be found. On the other hand, the “boundaries” of the BMI grouping themselves are optimization variables, as the partitioning method directly affects the global risk.

Optimal one-dimensional clustering combined with dynamic programming can be utilized. First, all pregnant women are sorted in ascending order of BMI to form an ordered sequence. The problem is then transformed into: partitioning the ordered sequence of length $\boldsymbol{\Phi }$ into $\boldsymbol{\Phi }$ contiguous segments (i.e., BMI groups) and selecting an optimal testing timepoint *W* for each segment to minimize the total risk.

Define $\boldsymbol{\phi }[i][j]$ as the cumulative risk achievable when the first *i* women (sorted by BMI) are partitioned into *j* groups. Assuming the last group contains women from index $p+1$ to *i*, then the first *p* women must form an optimal solution partitioned into $j-1$ groups. Therefore, $\boldsymbol{\phi }[i][j]$ can be computed by iterating over all possible split points *p*, with the state transition equation given by

15 $$\begin{aligned} \boldsymbol{\phi }[i][j]=\min _{j-1 \le p < i}\big \{\boldsymbol{\phi }[p][j-1]+\text {Cost}(p+1,i)\big \}, \end{aligned}$$

where $\text {Cost}(p+1,i)$ represents the minimized risk for a single group comprising the women from index $p+1$ to *i*.

To compute $\text {Cost}(p+1,i)$, it is necessary to find the optimal testing timepoint $W_{p+1,i}$ for this group, and then substitute it into the risk formula. The risk formula is

16 $$\begin{aligned} \text {Cost}(p+1,i)=\min _{W}\big \{(i-p)\cdot R_{p+1,i}(W)\big \}=(i-p)\cdot R_{p+1,i}(W_{p+1,i}), \end{aligned}$$

where $R_{p+1,i}(W)$ is the risk function for the group consisting of samples from $p+1$ to *i*.

### Multi-factor testing timepoint optimization model

When considering that the time for fetal Y-chromosome concentration to reach the threshold is influenced by various factors such as maternal height, weight, and age, it is necessary to comprehensively incorporate these factors, testing errors, and the proportion of fetuses reaching the threshold into the analysis. To address this, this study will employ a clustering algorithm to group pregnant women with similar detection data characteristics based on multiple physiological features. This approach aims to comprehensively reflect the impact of individual characteristics, gestational age, and model uncertainty on the likelihood of reaching the threshold. Building upon the Y-chromosome concentration prediction model (M4) established in the first research question and integrating a joint optimization approach, this study will simultaneously determine the optimal clustering groups and their corresponding best testing time points. The ultimate objective is to achieve the minimization of total global risk.

#### Cluster analysis

To comprehensively account for various maternal physiological characteristics, a clustering algorithm^17^ can be employed to group pregnant women with similar testing data profiles. Each woman corresponds to a *d*-dimensional feature vector $\boldsymbol{X}_i=(x'_{i1},x'_{i2},...,x'_{id})^T$, which includes metrics such as BMI, age, and weight. As the features have different scales and magnitudes, standardization is necessary before clustering. We applied the Z-score^18^ standardization method

17 $$\begin{aligned} x_{ij}=\frac{x'_{ij}-\mu _j}{\sigma _j},\quad i=1,2,...,N;\ j=1,2,...,d \end{aligned}$$

where $\mu _j$ and $\sigma _j$ represent the mean and standard deviation of the *j*-th feature across all pregnant women, respectively. The standardized feature vector is denoted as $\boldsymbol{X}_i=(x_{i1},x_{i2},...,x_{id})^T$, where each feature has a mean of 0 and a standard deviation of 1, thereby eliminating the influence of differing units.

After standardizing the core variables in the dataset, to directly clarify the relationships between various factors and Y chromosome concentration as well as gestational age, a multivariate scatter plot matrix was plotted using MATLAB.

**Fig. 3:**
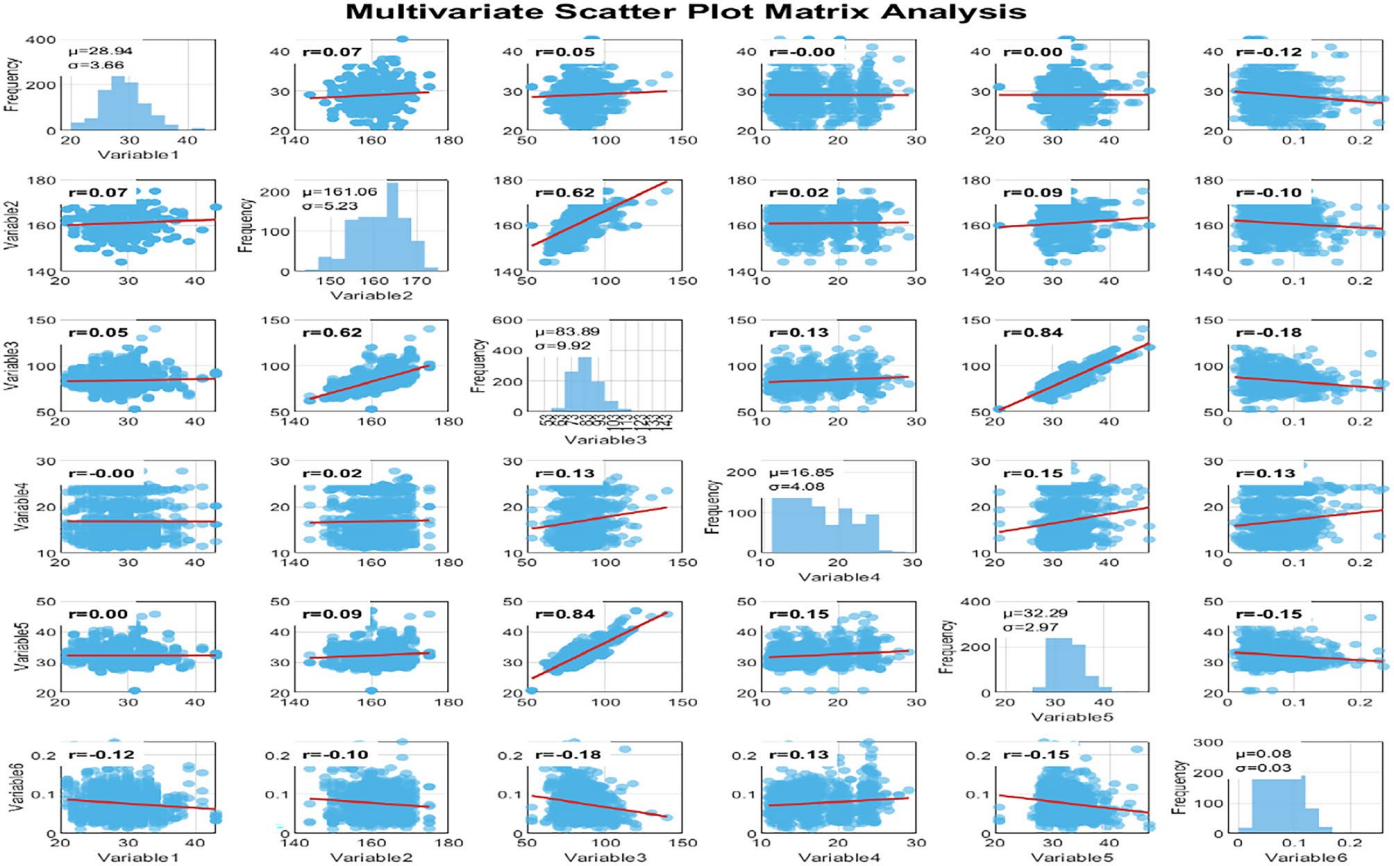
Scatter plot matrix of multiple factors.

Variables 1 to 6 in the Fig. 3 correspond to standardized age, standardized height, standardized weight, gestational age, standardized BMI, and standardized Y chromosome concentration in sequence. From the scatter plot, it can be concluded that: standardized BMI showed a weak negative correlation with Y chromosome concentration ($r = -0.15$), and high BMI mostly corresponded to Y chromosome concentration lower than 0.1%; Y chromosome concentration was concentrated in the range of 0.08 − 0.12% at 14−18 weeks of gestational age; height had a strong positive correlation with weight ($r = 0.62$); age had a weak association with other factors, and it is necessary to exert its effect through interaction terms.

Subsequently, the K-means clustering algorithm was employed to partition the standardized samples into *K* mutually exclusive groups $\{G_1,G_2,...,G_K\}$. The optimization objective of the algorithm is to minimize the within-cluster sum of squares, formulated as

18 $$\begin{aligned} \min _{\{\ G_k\}}=\sum _{k=1}^{K}\sum _{X_i \in G_k}\Vert \boldsymbol{x}_i-\mu _k\Vert ^2, \end{aligned}$$

where $\mu _k$ represents the centroid of the *k*-th group $G_k$. The number of clusters *K* can be determined using methods such as the elbow method or the silhouette coefficient.

Through cluster analysis^19^, pregnant women with similar physiological characteristics can be grouped into the same category, thereby laying the foundation for subsequently recommending a unified testing timepoint within each category.

#### Probability of success model

To comprehensively reflect the influence of individual maternal characteristics, gestational age, and model uncertainty on the likelihood of a successful test, a probability of success model is constructed based on the Y-chromosome concentration prediction model (5) ($M_4$) established in the first problem. The Y-chromosome concentration prediction model is given by

19 $$\begin{aligned} Y_i(w)=f(w,\boldsymbol{x}_i;\theta )+\delta _i. \end{aligned}$$

The factors such as gestational week, age, and height of the pregnant woman and is determined by the parameter set $\boldsymbol{\theta }$.

Therefore, the probability that the Y-chromosome concentration for the *i*-th pregnant woman reaches or exceeds the 4% threshold when tested at gestational week *W* can be expressed as

20 $$\begin{aligned} P_i(w)=\Phi \left( \frac{f(w,\boldsymbol{x}_i;\theta )-0.04}{\sigma }\right) , \end{aligned}$$

where $\Phi (\cdot )$ is the cumulative distribution function of the standard normal distribution.

#### Joint optimization model

The objective is to simultaneously determine the optimal clustering groups $\{G_k\}_{k}^{K-1}$ and the corresponding vector of optimal testing timepoints $\boldsymbol{w}=(w_1,w_2,...,w_K)^T$, in order to minimize the global total risk. Integrating risk formulas (10), (13), and (14), this joint optimization problem can be formulated as

21 $$\begin{aligned} \min _{\{G_k\},w}\sum _{k=1}^{K}|G_k|\cdot R_k(w_k), \end{aligned}$$

where *K* is the number of clusters, $\{G_k\}$ denotes the *k*-th cluster, and $|G_k|$ represents the number of pregnant women in the *k*-th group.

### Model for detecting chromosomal abnormalities in female fetuses

Since neither pregnant women nor female fetuses carry a Y-chromosome, accurately determining the presence of chromosomal abnormalities in female fetuses is critical. This study constructed a classification model targeting the aneuploidy status (columns A and B) of chromosomes 21, 18, and 13 using data from pregnancies with female fetuses. The model comprehensively incorporated the Z-scores, GC content, read counts, and relevant ratios of the X chromosome and the aforementioned autosomes, along with maternal BMI. First, the key features in the data were categorized into four groups: core chromosomal metrics, prediction quality indicators, maternal physiological characteristics, and pregnancy-related features. For feature selection, a dual screening strategy combining correlation analysis with feature importance ranking was adopted to eliminate redundant information. Subsequently, Decision Tree, Random Forest, and Support Vector Machine (SVM) models were built and compared for abnormality classification. The results indicate that the Support Vector Machine (SVM) demonstrated superior overall performance for this task.

#### Data preprocessing and outlier analysis

**Fig. 4:**
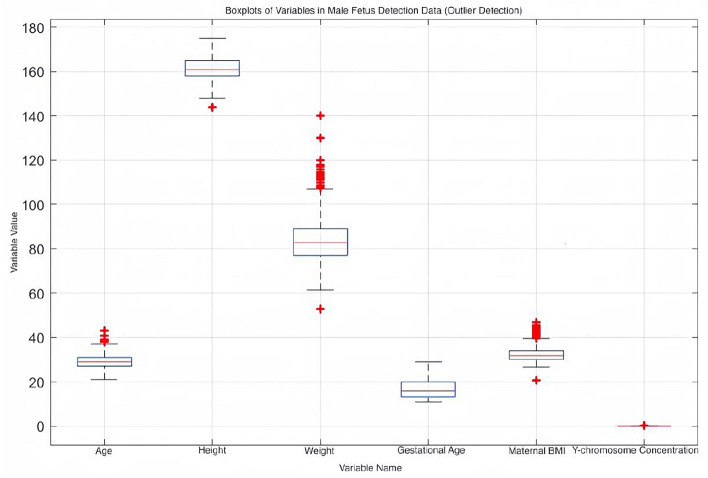
Box plots of various factors.

Integrating factors such as maternal height, weight, age, and BMI, along with detection errors and the proportion of cases meeting the Y-chromosome concentration threshold ($\ge 4\%$), the optimal NIPT timing was optimized through multi-factor clustering. Prior to this, data preprocessing was performed. Outliers were analyzed using the IQR method, and the resulting box plots are shown in Fig. 4.

Based on the box plots and statistical information in Fig. 4, the following observations were made. Gestational week at testing contained no outliers. Height had 4 outliers. Age, weight, BMI, and Y-chromosome concentration had 23, 29, 24, and 10 outliers, respectively. However, all fell within clinically reasonable ranges and thus were retained for subsequent standardization and clustering without removal.

To facilitate data processing, non-empty values in the “chromosomal aneuploidy” column were labeled as 1 (67 cases total), and empty values were labeled as 0 (538 cases total). Twenty key features were selected, including age, height, weight, maternal BMI, Z-scores for chromosomes 13, 18, 21, and X, X-chromosome concentration, GC content, raw read counts, etc. Missing values were imputed using the median of the respective column. Non-numeric entries such as “$\ge 3$” were replaced with the numeric value “3”. Subsequently, the data were standardized.

#### Feature categorization

Female fetuses lack a Y chromosome; therefore, the key to NIPT abnormality detection lies in identifying aneuploidies of chromosomes 21, 18, and 13. The detection process requires comprehensive consideration of X-chromosome related metrics, Z-scores of the target chromosomes (21, 18, 13), GC content, read count features, and individual maternal factors such as BMI. The input features were categorized into four groups.

- Core chromosomal indicators;
- Prediction quality metrics;
- Maternal physiological characteristics;
- Gestation-related features.

#### Key feature selection model

A dual-stage feature screening strategy, integrating correlation analysis and feature importance ranking^20^, was implemented to reduce data redundancy. First, the correlation coefficient (*r*) between each feature and the aneuploidy status of chromosomes 21, 18, and 13 was computed. Features with $|r| \ge 0.1$ were retained, while those weakly correlated ($|r| < 0.1$) were removed. Subsequently, the importance of the retained features was evaluated using a Random Forest model. An initial Random Forest model^21^ was built to calculate the “mean decrease in Gini impurity^22^” for each feature, which is formulated as

26 $$\begin{aligned} IM_j=\frac{1}{N}\sum _{t\in T}\Delta \text {Gini}_{j,t} , \end{aligned}$$

where $N=100$, *T* is the set of all decision trees, and $\Delta \text {Gini}_{j,t}$ is the reduction in the Gini impurity at a node where feature *j* is used for splitting in tree *t*. Finally, the top 15 features ranked by importance were retained.

#### Decision tree model for abnormality detection

A decision tree model^23^was constructed with “Aneuploidy of chromosomes 21/18/13” as the target. To prevent overfitting when sample sizes were small, the maximum depth was limited to 5, and the minimum number of samples required to split a node was set to 10. The Gini impurity was used as the criterion for node splitting, defined as

27 $$\begin{aligned} \text {Gini}(D)=1-\sum _{k=1}^{K}p_k^2 , \end{aligned}$$

where $p_k$ is the proportion of class *k* in node *D*. The optimal split was determined by minimizing the weighted Gini index.

#### Random forest model for abnormality detection

Building upon the decision tree, a Random Forest model was constructed, and its performance was evaluated with varying numbers of trees.

- With 50 trees: Mean Squared Error = 0.152 and $R^2$ = 0.783, indicating a relatively poor ensemble effect.
- With 150 trees: Mean Squared Error = 0.076 and $R^2$ = 0.911, showing slightly better performance but at the cost of significantly increased training time.
- With 100 trees: Mean Squared Error = 0.081 and $R^2$ = 0.902, which met the detection requirements while maintaining computational efficiency; therefore, this configuration (100 trees) was selected.

Each tree was trained on a bootstrap sample (70% of data) using a random subset of features. The final class for a sample was determined by majority voting of the predicted probabilities from all trees, reducing overfitting risk through ensemble learning. The mean decrease in Gini impurity was used to interpret feature contributions (e.g., the Z-score of chromosome 21 had the highest importance).

#### SVM model for abnormality detection

To handle the nonlinear relationship between features and the target variable, a Support Vector Machine^24^ (SVM) model with a Radial Basis Function (RBF) kernel was constructed. A “one-vs-one” strategy was employed, building 6 binary SVM sub-models, with the final class determined by a voting mechanism. The RBF kernel function is defined as

28 $$\begin{aligned} K(\textbf{x},\textbf{x}')=\exp \left( -\frac{\Vert \textbf{x}-\textbf{x}'\Vert ^2}{2\sigma ^2}\right) , \end{aligned}$$

where $K(\textbf{x},\textbf{x}')$ is the kernel function value (similarity) between samples $\textbf{x}$ and $\textbf{x}'$, $\Vert \textbf{x}-\textbf{x}'\Vert$ is the Euclidean distance, $\sigma$ is the kernel bandwidth parameter controlling the function width, and $\exp$ is the exponential function.

## Experimental results and analysis

### Results and analysis of the correlation model between gestational age, BMI, and Y-chromosome concentration

#### Performance comparison of the four models

To evaluate the performance of the different models, key metrics including $R^2$, log-likelihood, RMSE, and random effect variance were quantified and analyzed for models M1 through M4 as shown in Table 2.

**Table 2:** Comparison of fitting performance among the four candidate models.

Model	Model Type	$R^2$	Log-Likelihood	RMSE	Random Effect Variance
M1	Multiple linear regression	0.0335	1887.9059	0.0297	–
M2	Nonlinear regression	0.0350	1888.6060	0.0297	–
M3	Linear mixed-effects	0.7862	2127.7281	0.0140	0.0007
M4	Nonlinear mixed-effects	0.7891	2114.6934	0.0139	0.0007

**Fig. 5:**
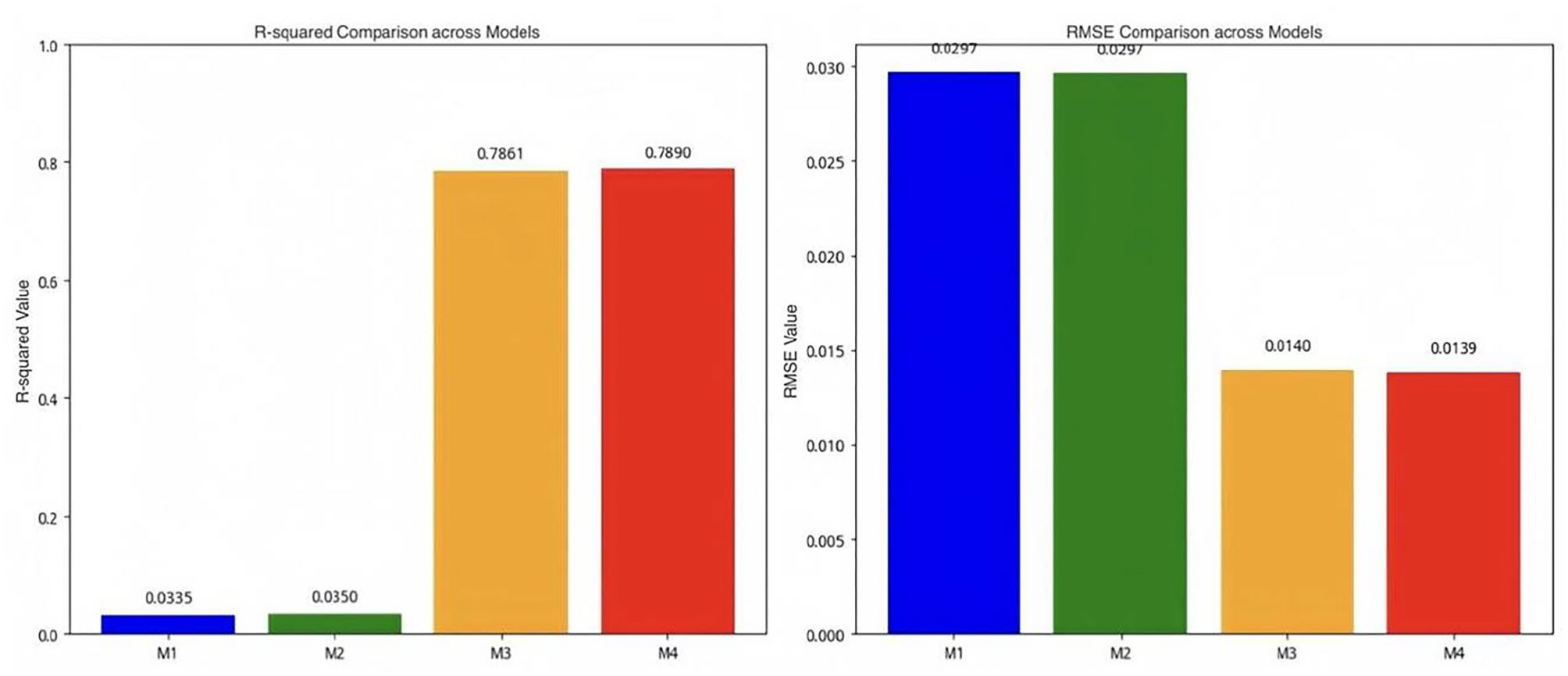
Comparison of fitting performance among the four models.

Figure 5 illustrates the comparison of fitting performance among models M1 to M4 in terms of $R^2$, log-likelihood, and RMSE.

To judge the relative merits of models M1 to M4, Fig. 4 shows that from M1 to M3, $R^2$ continuously improved, the log-likelihood increased to 2127.7281, and RMSE decreased to 0.014. After adjusting the fixed effects from a linear to a nonlinear relationship in M4, $R^2$ improved further, RMSE decreased to 0.0139, and although the log-likelihood slightly decreased, the random effect variance remained unchanged. This indicates that the nonlinear mixed-effects model more accurately captures the data characteristics. Therefore, M4 demonstrated the best performance across all metrics, offering superior fit, high accuracy, and reliable utility for both data description and prediction.

#### Fitting results and significance testing of the M4 model

After comparing the performance of the four candidate models from the perspectives of goodness-of-fit and statistical significance of parameters, the nonlinear mixed-effects model (M4) was ultimately selected as the optimal model for describing the relationship between fetal Y-chromosome concentration and related factors. To more intuitively analyze the patterns of fetal Y-chromosome concentration variation with gestational age and maternal BMI, as well as the characteristics of inter-individual differences among pregnant women, the average trend described by the model was overlaid with the raw data scatter points for visualization. The parameter estimation results of the model and the fitting of Y-chromosome concentration against gestational age and maternal BMI are presented as shown in Table 3.

**Table 3:** Parameter estimation results of the nonlinear mixed-effects model (M4).

Effect Type	Parameter	Coefficient estimate	Standard error	z-value	*P*-value
Fixed Effect	Intercept	0.198723	0.012456	15.953	$<0.000001$
Fixed Effect	Gestational Age	-0.029871	0.002134	-13.997	$<0.000001$
Fixed Effect	Gestational Age^2^	0.001985	0.000102	19.461	$<0.000001$
Fixed Effect	Gestational Age^3^	-2.987e-05	2.15e-06	-13.893	$<0.000001$
Fixed Effect	Maternal BMI	-0.004982	0.000512	-9.730	$<0.000001$
Random Effect	Maternal BMI^3^	7.985e-06	8.21e-07	9.726	$<0.000001$
Random Effect	Intercept: $\sigma ^2$	0.03198	0.01023	3.126	0.00177

To intuitively analyze the variation pattern of fetal Y-chromosome concentration with gestational age and maternal BMI, the average trend from the model was overlaid with the raw data scatter points for visualization.

**Fig. 6:**
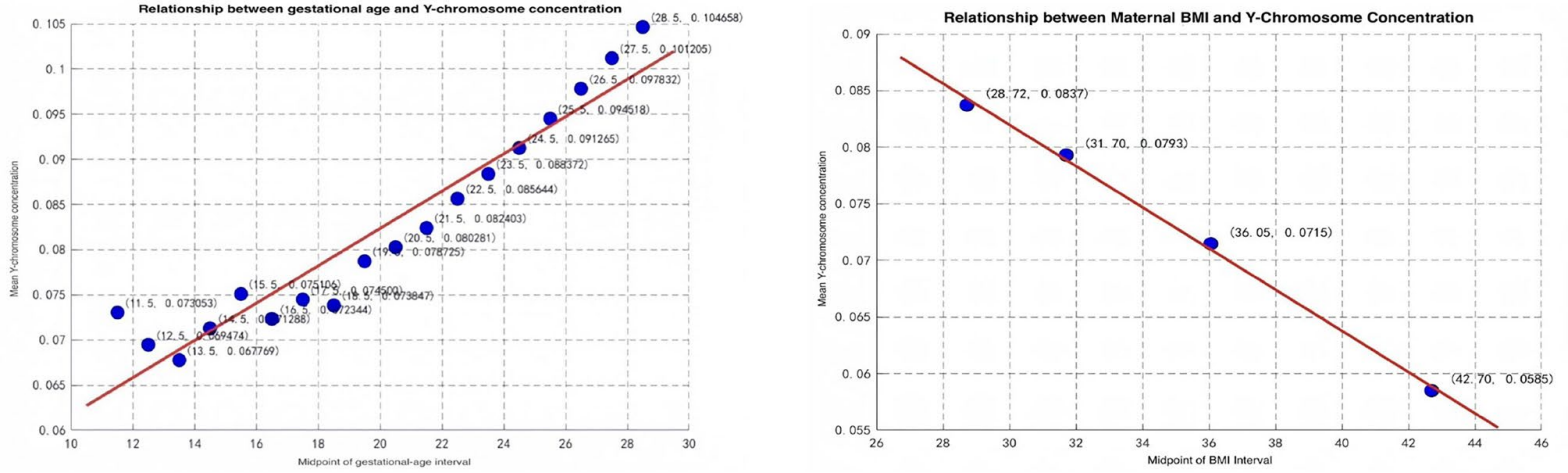
Fitting results of the final model.

Figure 6 shows that as the midpoint of the maternal BMI interval increases, the mean Y-chromosome concentration exhibits a decreasing trend, visually confirming the negative correlation between BMI and Y-chromosome concentration. As the midpoint of the gestational week interval increases, the Y-chromosome concentration shows an upward trend, indicating a positive correlation. Furthermore, the fitting results of Model M4 in Table 3 reveal that the coefficient estimate for the intercept is 0.198723, with a standard error of 0.012456 and a z-value of 15.953. This indicates the baseline Y-chromosome concentration when variables like gestational age and BMI are at theoretical reference levels. The coefficients for the linear, quadratic, and cubic terms of gestational age collectively support a cubic curve growth pattern of Y-chromosome concentration with gestational age: relatively slow growth in early pregnancy, acceleration in mid-pregnancy, and a slowdown in growth rate in late pregnancy. The coefficients for the linear and cubic terms of maternal BMI indicate a nonlinear negative relationship between BMI and Y-chromosome concentration.

The random effect focuses on the variance of the intercept $\sigma _u^2$ in Table 3. Its coefficient estimate is 0.03198, with a standard error of 0.01023, a z-value of 3.126, and $P<0.01$, indicating statistical significance. This suggests that beyond gestational age and BMI, maternal individual factors have a non-negligible modulating effect on Y-chromosome concentration. Regarding the overall model goodness-of-fit, as shown in Table 2, the conditional $R^2=0.7891$ explains approximately 78.91% of the variation in Y-chromosome concentration. The root mean square error (*RMSE*) is 0.0139, reflecting good predictive performance.

### Results and analysis of the joint optimization model for BMI stratification and testing timepoint

#### Model results and analysis

The core objective of this modeling approach is to “minimize the global expected total risk.” By integrating linear regression prediction, risk function construction, and dynamic programming optimization, the joint optimization of BMI stratification and the optimal NIPT timing was achieved through the following six steps.

**Step 1:** Initialize model parameters. Set the risk weight to 0.5, the Y-chromosome concentration threshold for the delay risk coefficient to 4%, the gestational week range for testing to [10, 25] with a step size of 0.1 weeks. The total variance is subsequently estimated through fitting.

**Step 2:** Fit a linear regression model using gestational age, BMI, and their interaction terms as independent variables. Calculate the residual standard deviation as the total variance and evaluate the model fit.

**Step 3:** Define and calculate the two types of risks. The test failure risk is based on the probability of concentration falling below the threshold, and the delay risk is calculated based on segmented gestational weeks. They are finally combined into a total risk function using the weight.

**Step 4:** Pre-compute the optimal testing timepoint for all possible groupings. Iterate over each BMI interval, calculate the total risk for different gestational weeks, and record the minimum risk and its corresponding gestational week to form a Cost matrix.

**Step 5:** Use dynamic programming to find the optimal stratification method. Iterate over possible split points based on sample count and number of groups, calculate cumulative risk, and update the minimum risk state and the corresponding grouping scheme.

**Step 6:** For each number of groups $(1\sim 5)$, extract the split points for dividing the total sample count into the corresponding number of groups from the auxiliary matrix. Determine the sample range for each group based on the split points, and calculate the BMI interval, sample size, and the optimal NIPT timepoint for that group extracted from the optimal gestational week matrix.

The algorithm based on the dynamic programming model was applied to a final dataset comprising 533 independent pregnant women. The model solution results are as follows in Table 4.

**Table 4:** Optimal NIPT timepoint results under different groupings.

Number of groups K	Group	BMI interval	Sample size	Optimal NIPT timepoint
1	1	[27.64, 46.88]	533	16.80
2	1	[27.64, 33.50]	271	15.20
2	2	[33.51, 46.88]	262	18.50
3	1	[27.64, 31.20]	185	14.50
3	2	[31.21, 36.80]	202	17.30
3	3	[36.81, 46.88]	146	20.10
4	1	[27.64, 29.80]	128	13.80
4	2	[29.81, 33.60]	156	16.10
4	3	[33.61, 38.50]	165	18.80
4	4	[38.51, 46.88]	84	21.50
5	1	[27.64, 29.00]	105	13.20
5	2	[29.01, 31.80]	132	15.50
5	3	[31.81, 35.20]	148	17.70
5	4	[35.21, 39.80]	112	19.90
5	5	[39.81, 46.88]	36	22.30

Analysis of the data in Table 4 indicates a positive correlation between maternal BMI and the optimal NIPT timepoint. As *K* increases, the global minimum cumulative risk gradually decreases, showing a diminishing marginal effect. $K = 3 \sim 4$ represents the optimal balance. When $K=5$, the sample size in the high-BMI group is only 36, resulting in low clinical management efficiency and insufficient cost-effectiveness. Balancing personalized grouping granularity with the statistical representativeness of within-group sample sizes, and avoiding bias from overly broad groupings with large BMI spans, the 4-group BMI stratification scheme for NIPT testing is recommended.

**Fig. 7:**
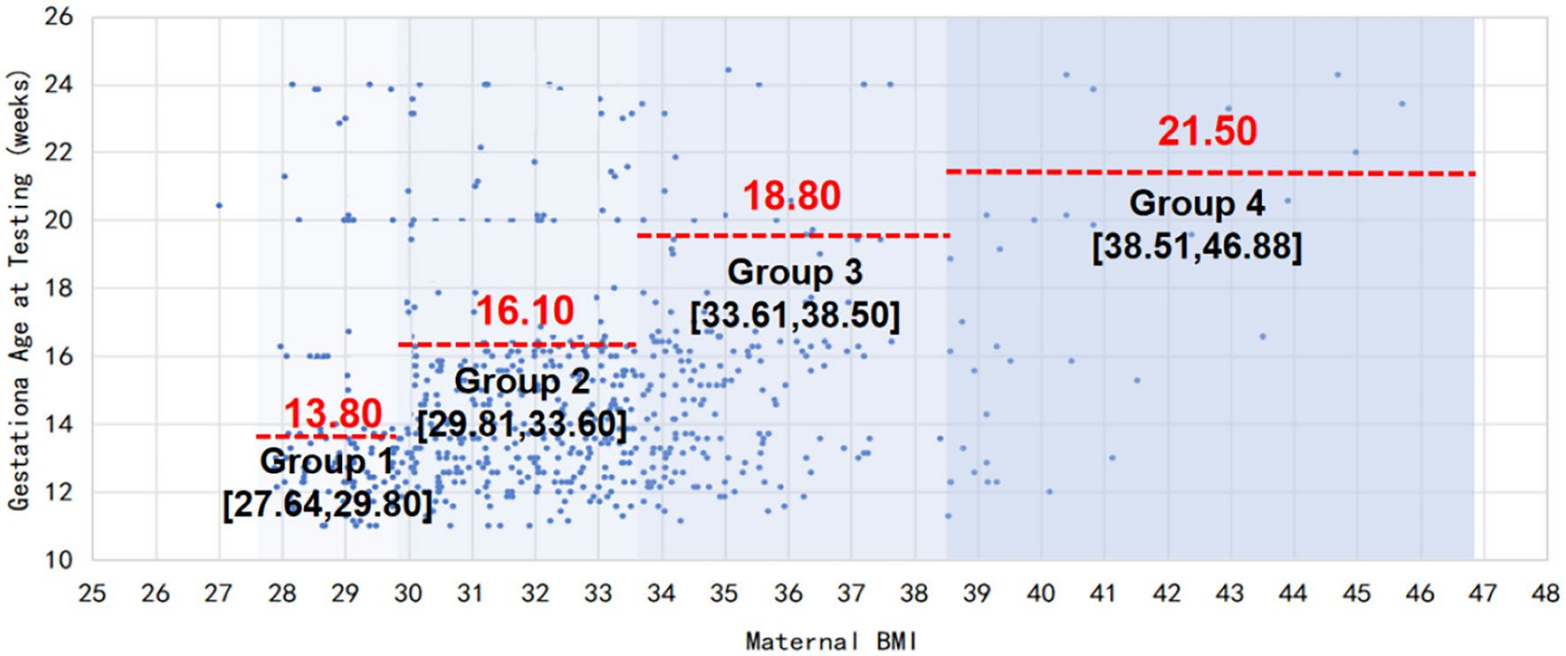
Decision map for BMI grouping and optimal NIPT timepoint (Scheme 4).

To visually corroborate the data distribution and risk changes under Scheme 4, a decision map was created by overlaying the BMI groups and their optimal NIPT time points from Scheme 4 onto a scatter plot of the real independent pregnant women sample data, as shown in Fig. 7.

From Fig. 7, it can be observed that the optimal testing time shifts to later gestational weeks as the BMI interval increases. When BMI is divided into four groups, the range of each group aligns more closely with the inherent distribution of the data, and the samples within each group are both sufficiently numerous and relatively evenly distributed. Considering the need to accurately reflect individual differences while meeting practical clinical requirements, the 4-group scheme was ultimately selected.

#### Analysis of the impact of testing error

**Fig. 8:**
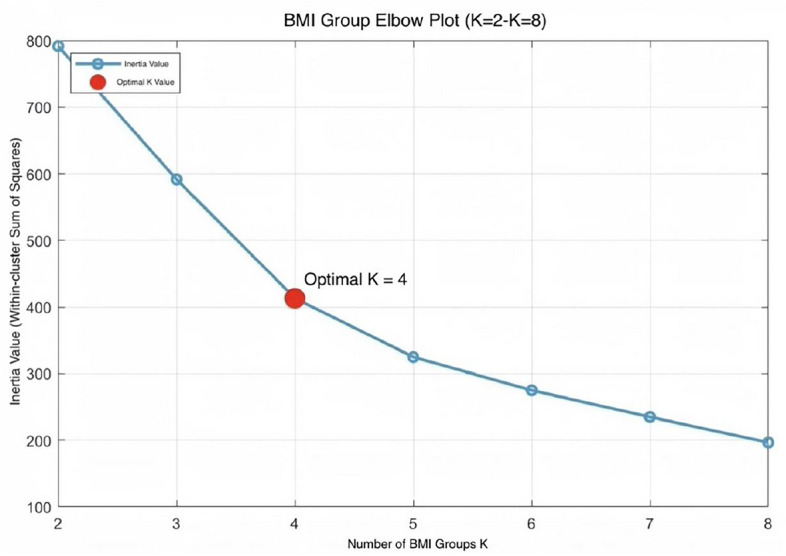
K-means elbow plot.

To further quantitatively validate the scientific basis of the $K=4$ grouping in Scheme 4 and analyze the impact of testing error on the results, this study employed the K-means^25^ elbow plot for analysis. By measuring the change in the sum of squared distances from samples to their cluster centers (inertia) with the number of clusters *K*, the optimal number of clusters was determined objectively to avoid the subjectivity of empirical grouping. The visualization result is as follows in Fig. 8.

The K-means elbow plot shows that the within-cluster sum of squares monotonically decreases with increasing *K*, and the rate of decrease slows down. $K=4$ is identified as the elbow point. Testing error only slightly affects clustering stability and group boundaries, without changing the conclusion that $K=4$ is the optimal grouping scheme.

In the analysis of testing error impact for Model 2, the prediction residuals of Y-chromosome concentration are central. The residual standard deviation $\sigma _{\text {test}}$ and the error amplification factor *k* were used to quantify the error and analyze its impact on risk and optimal NIPT timing. The data reconstruction involved features from 533 pregnant women samples. Five error scenarios covering a range of precision changes were defined. The optimal NIPT timepoint for each group under Scheme 4, along with risk metrics and global risk, were recalculated. The results are presented in Table 5.

**Table 5:** Risk metrics and global risk results.

Scenario	Group	BMI Interval	N	Original	Adjusted	Test Failure	Delay	Group Total	Global risk
				Timepoint	Timepoint	Risk	Risk	Risk	
S0 (k=0.8)	1	[27.64,29.80]	128	13.80	13.50	0.082	0.135	0.1085	0.121
S0 (k=0.8)	2	[29.81,33.60]	156	16.10	15.80	0.095	0.158	0.1265	0.121
S0 (k=0.8)	3	[33.61,38.50]	165	18.80	18.30	0.112	0.264	0.1880	0.121
S0 (k=0.8)	4	[38.51,46.88]	840	21.50	20.90	0.138	0.345	0.2415	0.121
S1 (k=1.0)	1	[27.64,29.80]	128	13.80	13.80	0.105	0.138	0.1215	0.143
S1 (k=1.0)	2	[29.81,33.60]	156	16.10	16.10	0.123	0.161	0.1420	0.143
S1 (k=1.0)	3	[33.61,38.50]	165	18.80	18.80	0.148	0.264	0.2060	0.143
S1 (k=1.0)	4	[38.51,46.88]	840	21.50	21.50	0.175	0.345	0.2600	0.143
S2 (k=1.2)	1	[27.64,29.80]	128	13.80	14.20	0.132	0.148	0.1400	0.168
S2 (k=1.2)	2	[29.81,33.60]	156	16.10	16.50	0.155	0.175	0.1650	0.168
S2 (k=1.2)	3	[33.61,38.50]	165	18.80	19.40	0.186	0.282	0.2340	0.168
S2 (k=1.2)	4	[38.51,46.88]	840	21.50	22.20	0.218	0.366	0.2920	0.168
S3 (k=1.5)	1	[27.64,29.80]	128	13.80	14.70	0.178	0.161	0.1695	0.205
S3 (k=1.5)	2	[29.81,33.60]	156	16.10	17.20	0.202	0.213	0.2100	0.205
S3 (k=1.5)	3	[33.61,38.50]	165	18.80	20.30	0.242	0.309	0.2755	0.205
S3 (k=1.5)	4	[38.51,46.88]	840	21.50	23.50	0.285	0.405	0.3450	0.205
S4 (k=2.0)	1	[27.64,29.80]	128	13.80	15.30	0.245	0.177	0.2110	0.258
S4 (k=2.0)	2	[29.81,33.60]	156	16.10	18.10	0.282	0.243	0.2625	0.258
S4 (k=2.0)	3	[33.61,38.50]	165	18.80	21.50	0.328	0.345	0.3365	0.258
S4 (k=2.0)	4	[38.51,46.88]	840	21.50	25.10	0.376	0.453	0.4145	0.258

From Table 5, it can be concluded that as the error level increases, the expected test failure risk for each group rises, which requires delaying the optimal NIPT time point to balance the risks. Under scenario S4, the global total risk increased by 32% compared to the baseline scenario S1, and the optimal time point was delayed by an average of 1.2 weeks. Scenario S0 could reduce the total risk by 18% with the time point advancing by 0.5 weeks, indicating that detection accuracy significantly affects the model results. In clinical practice, lower error may allow for appropriately advancing the test time point, while higher error requires strengthened quality control to avoid exceeding the recommended testing window. The optimal time point under the baseline scenario aligns with clinical guidelines, validating the rationality of the original scheme.

### Results and analysis of the multi-factor testing timepoint optimization model

Using SPSS, after eliminating scale differences among the standardized clustering variables, the elbow method was applied repeatedly in multiple runs of K-means clustering, ultimately determining the elbow point at $K=4$. Clustering was re-run to obtain cluster centers and group sample sizes. MATLAB was then used to solve the joint optimization model, yielding the coefficient results for the success probability model, as shown in Table 6.

**Table 6:** Coefficient results of the success probability model.

Variable	Coefficient estimate	Standard error	Z-value	*P*-value
Intercept	$-2.158$	0.321	$-6.723$	$<0.001$
Age	0.028	0.011	2.545	0.011
Standardized Height	0.185	0.072	2.569	0.010
Standardized Weight	$-0.152$	0.068	$-2.235$	0.025
BMI	$-0.053$	0.014	$-3.786$	$<0.001$
Gestational Age	0.087	0.021	4.143	$<0.001$
BMI$\times$Gestational Age	$-0.002$	0.001	$-2.018$	0.043

According to the results in Table 6, when BMI increases by 1 kg/m^2^, the success probability decreases by 5.3%; for each additional week of gestational age, the success probability increases by 8.7%; taller height and lower weight are associated with higher success rates. Furthermore, there is an interaction between BMI and gestational age, where the enhancing effect of gestational age on success probability weakens in the high-BMI group.

Based on the success probability model, combined with the total risk function comprising test failure risk and delayed diagnosis risk, the optimal time point and risk for each group were solved using the simulated annealing algorithm in MATLAB. The final results of the joint optimization model are presented in Table 7.

**Table 7:** Optimal timepoint and risk results.

BMI group	Optimal timepoint (weeks)					S1 Min. Risk
	S0	S1	S2	S3	S4	
Low BMI [20.64, 27.80]	13.5	13.8	14.2	14.7	15.3	0.122
Medium-Low BMI [27.81, 29.60]	15.5	16.1	16.5	17.2	18.1	0.142
Medium-High BMI [29.61, 32.50]	18.0	18.8	19.4	20.3	21.5	0.206
High BMI [32.51, 46.88]	20.6	21.5	22.2	23.5	25.1	0.260

According to Table 7, it can be concluded that for every 20% increase in detection error, the optimal timing is delayed by an average of 0.5–0.7 weeks. There are significant differences in the optimal timing and detection risk among different BMI groups. The low BMI group has the earliest optimal timing, the lowest minimum risk value under the S1 scenario, and the lowest sensitivity to detection error. The optimal timing for the medium-low and medium-high BMI groups falls between that of the low and high BMI groups, while the high BMI group has the latest optimal timing, the highest minimum risk value under the S1 scenario, and the largest shift in timing with error, indicating the highest sensitivity to detection error. These results show that the optimal detection timing for each BMI group is positively correlated with the S1 scenario; that is, the higher the BMI value, the later the optimal timing, the higher the baseline detection risk, and the lower the tolerance to error. The multi-factor optimal timings are finally determined as follows: low BMI group [20.64, 27.80] at 13.8 weeks, medium-low BMI group [27.81, 29.60] at 16.1 weeks, medium-high BMI group [29.61, 32.50] at 18.8 weeks, and high BMI group [32.51, 46.88] at 21.5 weeks.

### Results and analysis of the model for detecting chromosomal abnormalities in female fetuses

Models were implemented using the sklearn library, with “test set performance” as the core evaluation criterion. Considering the imbalanced data characteristics, focus was placed on overall discriminative ability, recall for the abnormal class, and precision for the abnormal class. The results are presented in Table 8.

**Table 8:** Test performance comparison.

Model	Acc	AUC	Abn Recall	Abn Prec	FN	FP
Decision Tree	0.775	0.521	0.296	0.267	19	22
Random Forest	0.901	0.725	0.800	0.533	4	14
SVM	0.852	0.807	0.550	0.393	9	17

Based on Table 8, the performance of the three models was observed. Random Forest achieved the best accuracy, while the Support Vector Machine (SVM) performed better in terms of the AUC metric. Therefore, SVM demonstrates stronger capability in distinguishing between anomaly categories.

**Fig. 9:**
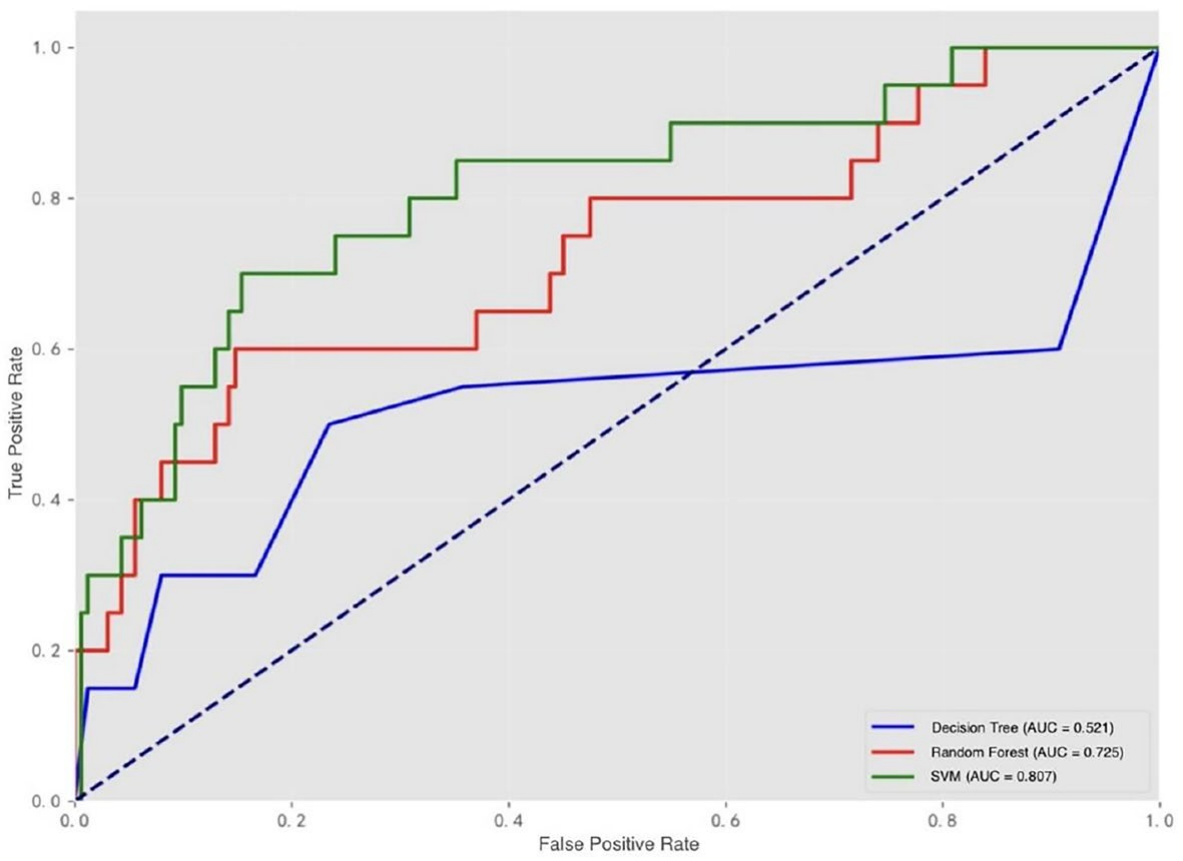
Comparison of ROC curves for the three classification methods.

The ROC^26^ curve (Fig. 9) also shows that SVM’s curve is closest to the top-left corner, followed by Random Forest, with Decision Tree performing the worst. This result suggests SVM’s advantage on imbalanced datasets.

**Fig. 10:**
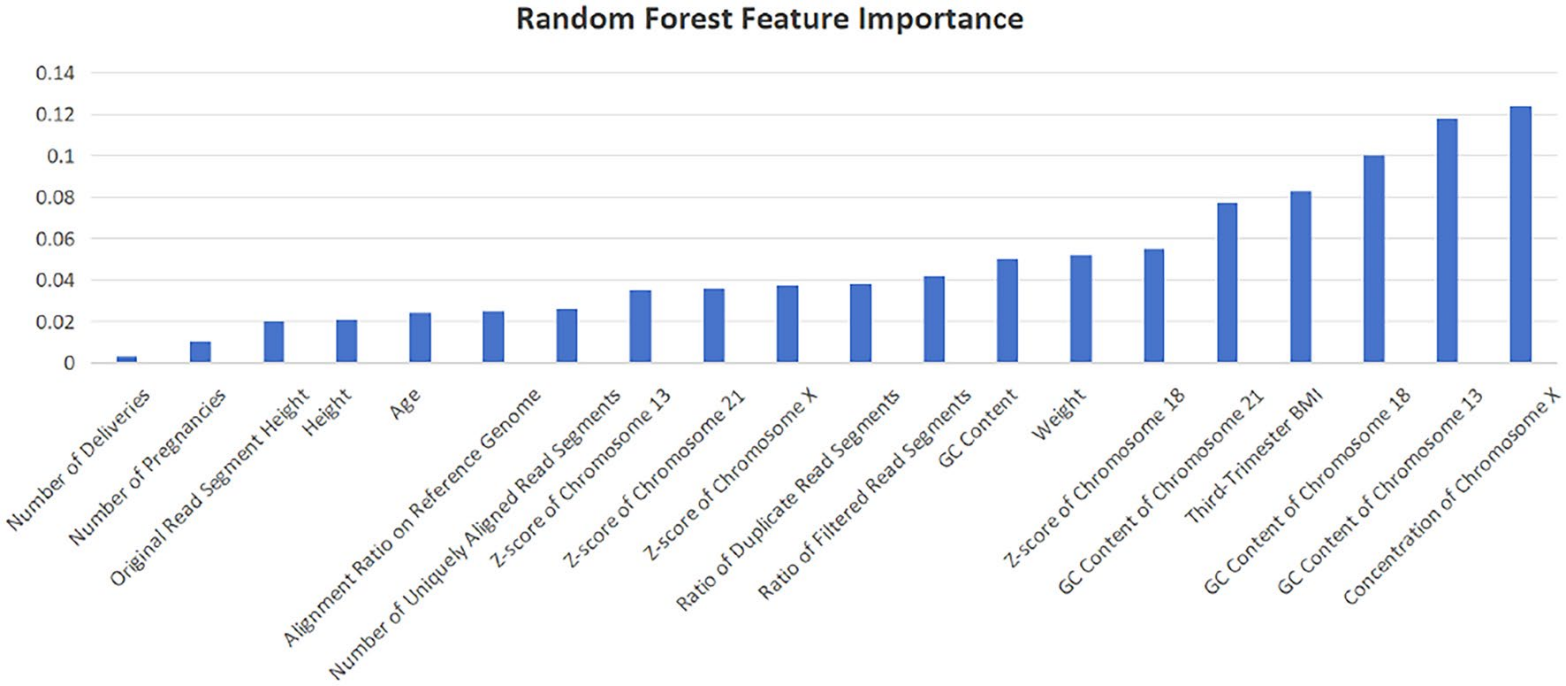
Random forest feature importance ranking.

A Random Forest feature importance ranking plot was constructed, as shown in Fig. 10, which visually illustrates that X-chromosome concentration and GC content are the dominant factors, followed by Z-scores. BMI and weight also show significant influence, suggesting these indicators should be prioritized for monitoring.

**Fig. 11:**
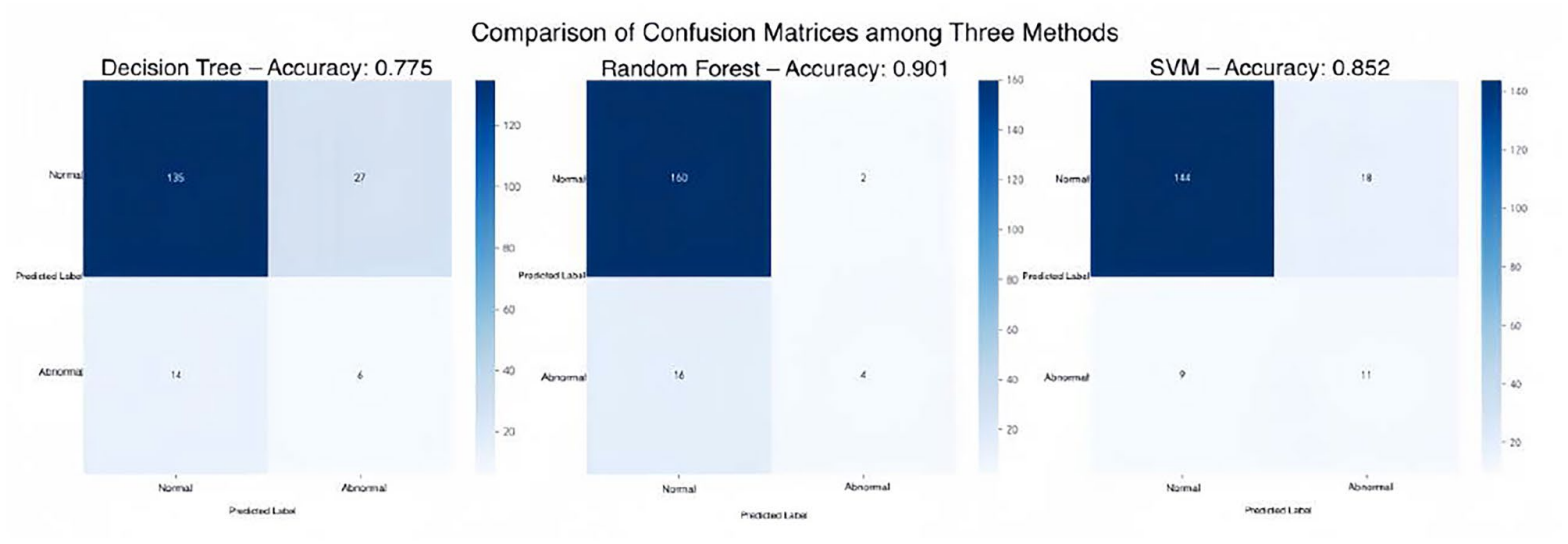
Comparison of confusion matrices for the three methods.

From the comparison of confusion matrices (Fig. 11), it can be seen that Random Forest has the lowest false-negative rate but a relatively high false-positive rate. In contrast, SVM exhibits a better balance across various metrics.

**Fig. 12:**
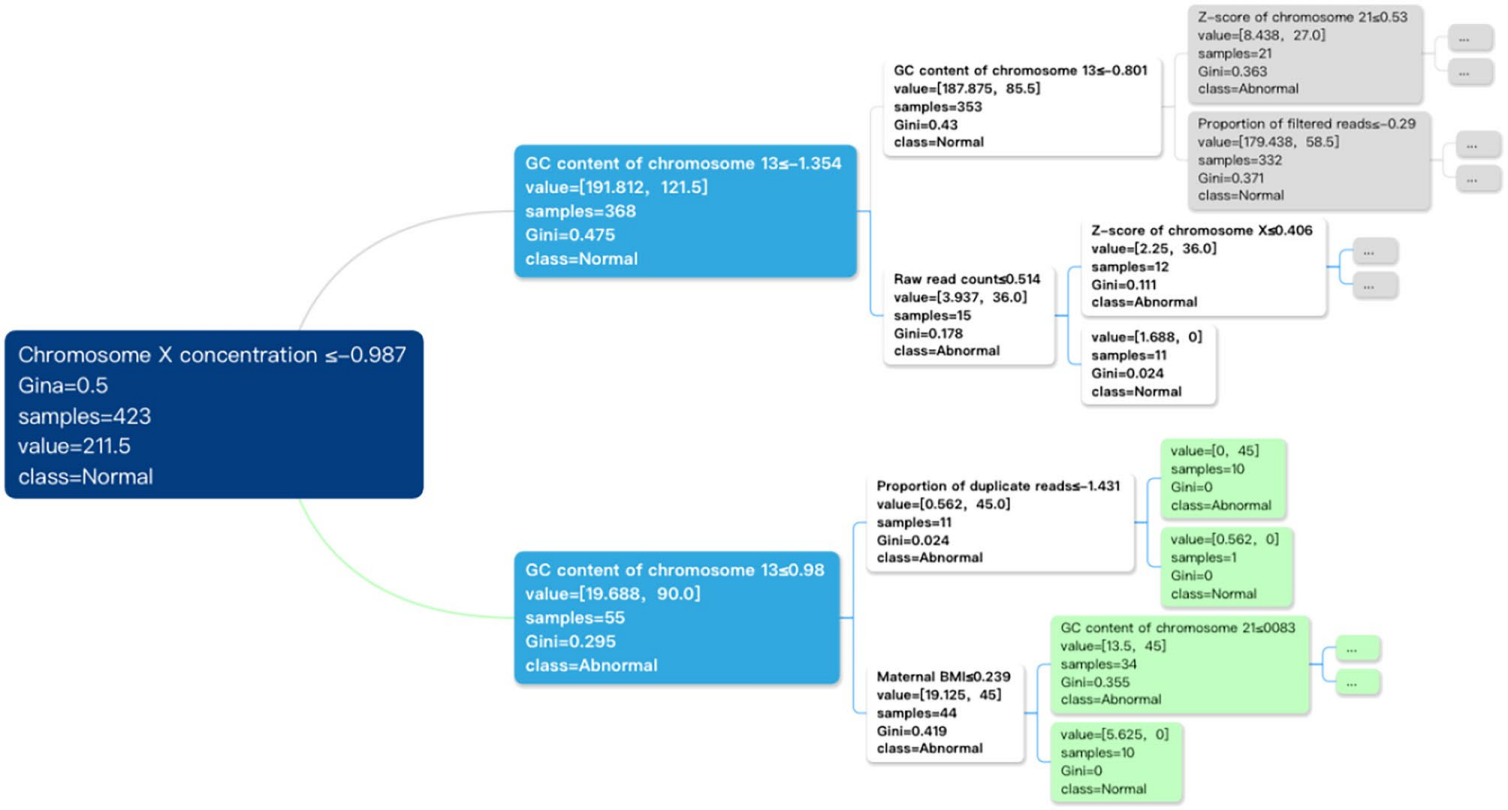
Decision tree structure diagram (First 3 Levels).

According to the decision tree structure diagram and rules (Fig. 12), when the X chromosome concentration is less than or equal to −0.987 and the GC content of chromosome 13 exceeds the threshold of −1.354, it will enter the branch path where the abnormal probability increases. Subsequently, after being filtered by conditions such as the original read segment number and the proportion of repeated read segments at the sub-nodes, the abnormal probability will increase significantly in the end.

**Fig. 13:**
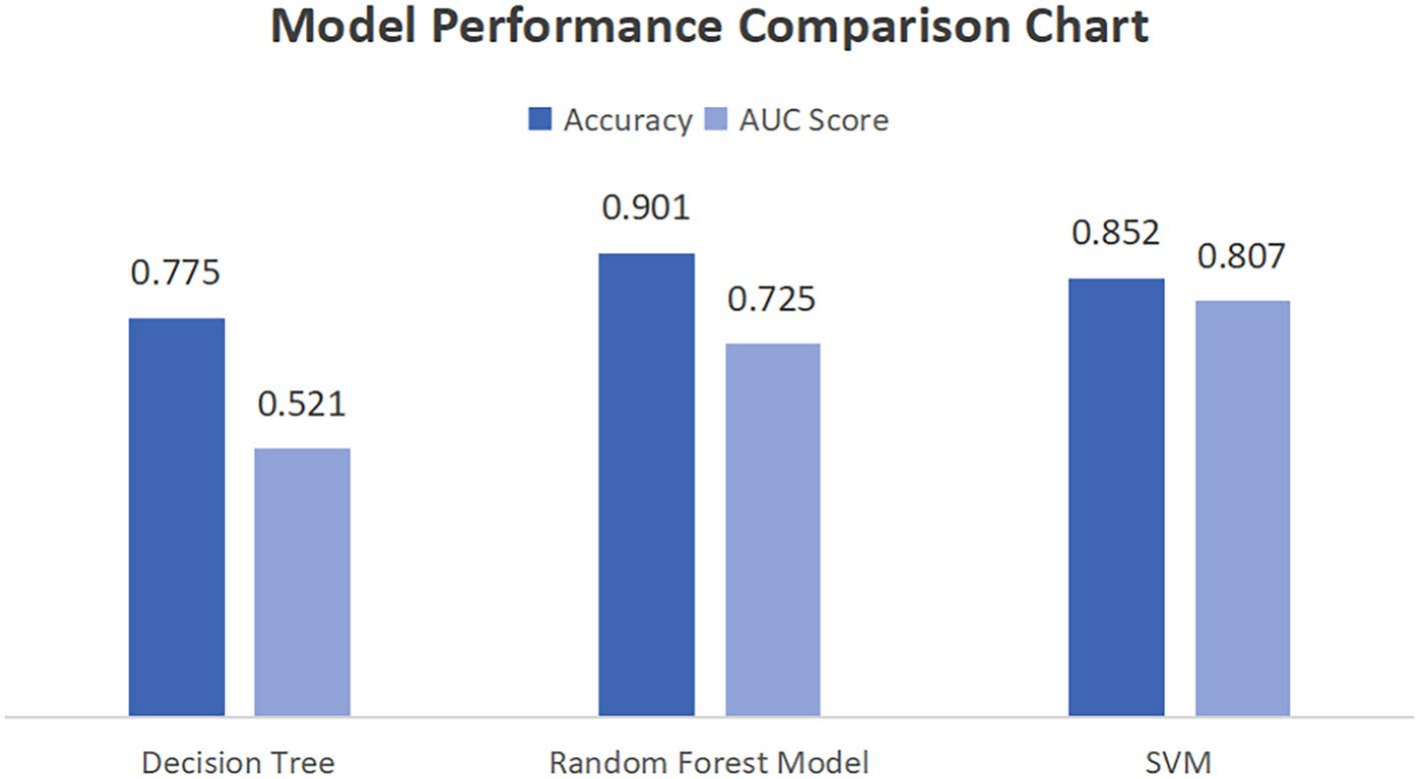
Model performance comparison.

From Fig. 13, by comparing the accuracy and AUC scores of the models, it is clear that SVM scores higher on both metrics compared to the Decision Tree and Random Forest models. Therefore, SVM is recommended as the primary model.

Based on the combined analysis, the judgment method should first examine the Z-scores; if the absolute value is greater than 3, it is considered abnormal. Then check if the GC content is within the range of 0.38 to 0.41, followed by examining the read count proportion to ensure a uniquely mapped rate exceeding 80%. Finally, input the data into the model to calculate the probability. If the result exceeds 0.6, a re-test is recommended. In clinical practice, it is advisable to first use the SVM model and then adjust the judgment criteria by incorporating BMI values to improve detection sensitivity.

## Discussion

In this study, the nonlinear mixed-effects model^27^ (M4) with $R^2=0.7891$ and $\text {RMSE}=0.0139$ demonstrates its effectiveness in explaining the association between gestational age, BMI, and Y-chromosome concentration, concluding that “BMI is nonlinearly negatively correlated with Y-chromosome concentration.” This quantifies the dilution effect of high BMI on the fetal fraction (FF)^28^. The gradient of testing timepoints (13.80-21.50 weeks) corresponding to the 4-group BMI scheme clarifies the timing differences for different weight groups, providing actionable indicators for precise clinical timing selection. The mechanism behind the delayed optimal testing timepoint for high-BMI pregnant women is essentially a physiological chain reaction triggered by increased BMI: as BMI rises, the total amount of cell-free DNA in maternal peripheral blood increases correspondingly, which indirectly reduces the proportion of fetal DNA within it^29^. Since the fetal fraction itself increases slowly with gestational age in early pregnancy, high-BMI groups require a longer gestation period to accumulate sufficient fetal DNA to meet testing requirements, necessitating a corresponding delay in their testing timepoint. The SVM model performs better in detecting abnormalities in female fetuses because its nonlinear fitting of multiple features (X-chromosome concentration, GC content) compensates for the limited discriminatory power of single indicators for female fetal chromosomal abnormalities. The combined results indicate that the “BMI stratification + individualized testing timepoint” scheme can effectively improve NIPT accuracy in high-BMI groups, while the SVM multi-feature integration system can enhance the efficacy of abnormality detection in female fetuses. Together, they address the current shortcomings of NIPT in special populations and gender-specific determination.

The main strength of this study lies in achieving individualized matching of testing time points through BMI stratification. Both the model and the proposed strategy are constructed based on empirical data from a large sample (1,687 cases), indicating good potential for clinical translation. The current model is applicable for the detection of trisomy abnormalities in chromosomes 21, 18, and 13.

## Conclusion

This study addressed the core issues in the clinical application of NIPT: “test failure due to individual differences” and “limited dimensions for detecting abnormalities in female fetuses^30^.” By employing methods such as BMI stratification analysis, the nonlinear mixed-effects model (M4), and SVM multi-feature integration, the study systematically explored the optimal NIPT testing timepoints for different BMI intervals and constructed a detection system for chromosomal abnormalities in female fetuses. The results indicate a significant positive correlation between BMI and the optimal NIPT testing timepoint (higher BMI requires later testing). The 4-group BMI scheme (intervals: [27.64, 29.80], [29.81, 33.60], [33.61, 38.50], [38.51, 46.88]) with corresponding optimal testing timepoints (13.80, 16.10, 18.80, 21.50 weeks) can effectively improve detection accuracy in high-BMI groups. Simultaneously, the SVM model achieved an AUC^31^ of 0.807 in detecting female fetal abnormalities, significantly outperforming traditional single-indicator methods. The “BMI-testing timepoint” individualized scheme proposed in this study can provide quantitative references for clinical NIPT screening, contributing to the precision development of prenatal screening. Future work could expand the sample scope, include confounding factors such as age and comorbidities to build a multi-dimensional integrated model, and further optimize the scheme’s applicability and reliability through prospective clinical validation.

## Supplementary Information

Below is the link to the electronic supplementary material.

Supplementary Material 1

## Data Availability

The dataset used in this study is derived from Problem C of the 2025 Higher Education Society Cup National College Student Mathematical Modeling Competition (China). The original dataset is available in the Zenodo repository under the DOI: 10.5281/zenodo.19276819.
